# Synthesis, Characterization, and Biological Evaluation of Anti-HER2 Indocyanine Green-Encapsulated PEG-Coated PLGA Nanoparticles for Targeted Phototherapy of Breast Cancer Cells

**DOI:** 10.1371/journal.pone.0168192

**Published:** 2016-12-12

**Authors:** Yu-Hsiang Lee, Yun-Han Lai

**Affiliations:** 1 Department of Biomedical Sciences and Engineering, National Central University, Taoyuan City, Taiwan R.O.C.; 2 Department of Chemical and Materials Engineering, National Central University, Taoyuan City, Taiwan R.O.C.; Univerzitet u Beogradu, SERBIA

## Abstract

Human epidermal growth factor receptor 2 (HER2)-overexpressed breast cancer is known to be more aggressive and resistant to medicinal treatment and therefore to whom an alternative therapeutics is needed. Indocyanine green (ICG) has been widely exploited in breast cancer phototherapy. However, drawbacks of accelerated degradation and short half-life (2–4 min) in blood seriously hamper its use in the clinic. To overcome these challenges, an anti-HER2 ICG-encapsulated polyethylene glycol-coated poly(lactic-co-glycolic acid) nanoparticles (HIPPNPs) were developed in this study. Through the analyses of degradation rate coefficients of ICG with and without polymeric encapsulation, the photostability of HIPPNP-entrapped ICG significantly enhanced 4 folds (*P* < 0.05) while its thermal stabilities at 4 and 37°C significantly enhanced 5 and 3 (*P* < 0.05 for each) folds, respectively, under equal lighting and/or heating treatment for 48 h. The target specificity of HIPPNPs to HER2-positive cells was demonstrated based on a 6-fold (*P* < 0.05) enhancement of uptake efficiency of HIPPNPs in MDA-MB-453/HER2(+) cells within 4 h as compared with that in MCF7/HER2(-) cells. Moreover, the HIPPNPs with ≤ 25 μM ICG equivalent were nontoxic to cells in the absence of light illumination, and enabled to generate similar amount of singlet oxygen and hyperthermia effect as compared with that used by free ICG upon NIR irradiation. After 808 nm-laser irradiation with intensity of 6 W/cm^2^ for 5 min, the viability of MDA-MB-453 cells pre-treated by HIPPNPs with ≥ 5 μM ICG equivalent for 4 h significantly reduced as compared with that treated by equal concentration of free ICG (*P* < 0.05) and > 90% of the cells were eradicated while the dose of HIPPNPs was increased to 25 μM ICG equivalent. In summary, the developed HIPPNPs are anticipated as a feasible tool for use in phototherapy of breast cancer cells with HER2 expression.

## Introduction

According to the statistics of World Health Organization, breast cancer is the most frequently diagnosed cancer and the leading cause of cancer death among females worldwide [1]. Currently breast cancer is commonly treated by chemo-, radio-, immuno-, and/or hormone- therapies in addition to surgery. Although the therapeutics of breast cancer is increasingly advanced over the past decades, metastatic breast cancer remains incurable and the 5-year overall survival rate is only 23% [2], implicating that an effective therapeutic strategy is still urgently needed. Among various types of breast cancer, the one with overexpression of anti-human epidermal growth factor receptor 2 (HER2) which is in nearly 30% of breast cancer located in either primary tumors or metastatic sites [3] has gained increasing attention in the last decade since its level is strongly correlated with breast cancer pathology including tumorigenesis, oncogenic transformation, metastasis, and poor prognosis [4–7]. Furthermore, the HER2-positive breast cancer is known to be more aggressive and resistant to medicinal treatment [7–9], implicating that improving the method of tumor destruction instead of changing anti-cancer drugs persistently may truly cure the HER2-positive breast cancer.

Among various approaches of breast cancer treatment, near-infrared (NIR)-mediated phototherapy is one of the most promising strategies for serving as a supplement to traditional cancer therapies since it can provide 1) enhanced tissue penetration efficacy as compared with that operated by visible light [10] and 2) moderate toxicity to normal cells/tissues through use of targeted photosensitive agents and/or spatially controlled light irradiation [11]. Generally speaking, phototherapy is carried out by hyperthermia and/or reactive oxygen species (ROS) generated from the photosensitizers under light illumination in the presence of oxygen that the former may cause thermal ablation of cancer cells (i.e., photothermal therapy; PTT) [11], while the latter may seriously interfere cellular metabolism and thus trigger programed cell death (i.e., photodynamic therapy; PDT) [11–13]. No matter which mechanism is utilized, the photosensitizer plays a key role in the effectiveness of phototherapy.

Indocyanine green (ICG) is an U.S. Food and Drug Administration (FDA)-approved tricarbocyanine dye which enables to absorb and fluoresce in the region of 650–850 nm. Currently, in addition to serving as a fluorophoric agent for use in diagnostic purposes such as NIR image-guided oncologic surgery [14], fluorescence angiography [15], and lymph node detection of cancer [16], ICG has been exploited as a photosensitizer for use in cancerous phototherapy including breast, brain, and skin tumors [17–19] since it enables to produce heat and ROS (i.e., singlet oxygen) upon NIR irradiation. Although ICG is of particular advantage for use in cancer phototherapy, it adversely tends to disintegrate in aqueous medium and such degradation can be markedly accelerated by light irradiation (photodegradation) and/or heating (thermal degradation) [20]. Furthermore, ICG after administered intravenously will be readily bound with blood proteins and hence leads to only 2–4 min of plasmatic half-life [21,22]. These circumstances seriously hinder the applicability of ICG in the clinic and thus a strategy that enables to enhance the aqueous stability and target efficiency of ICG is certainly needed for ICG-mediated therapy.

Nanomedicine may offer a feasible means for usage of ICG without aforementioned defects since it may provide merits of enhanced bioavailability, improved stability, and security for the payload [23]. In terms of the materials used for making drug carrier, polymer is often considered as the preferred candidate since it can be manipulated to tailor the properties and/or functionalities required by the product [24]. Among various pharmaceutical polymers, poly(lactic-co-glycolic acid) (PLGA) is the copolymer of poly(lactic acid) and poly(glycolic acid) and is one of the best defined biomaterials with FDA approval for drug encapsulation due to its biocompatibility, biodegradability, and controllability for drug release [25]. Polyethylene glycol (PEG), another FDA-approved polymer with characteristics of nontoxicity and less immunogenicity, is frequently used for surface modification of drug carrier since the retention time of the PEG-coated particle in the blood circulation can be markedly increased [26].

Taken all together, we aim to develop an anti-HER2 ICG-encapsulated PEG-coated PLGA nanoparticles (HIPPNPs) for targeted phototherapy of HER2-expressing breast cancer cells. We anticipate that the use of ICG by implantation of HIPPNPs instead of naked molecules is advantageous because the polymeric carrier (i.e., HIPPNP) may 1) potentially protect the entrapped ICG from degradation caused by external stimuli such as light, heat, and/or extreme pH [20,27]; 2) preciously localize the therapeutic region to reduce off-target toxicity, and 3) provide accurate estimation for the efficacy of ICG-mediated phototherapy. In this study, the fabrication procedures of HIPPNPs was first detailedly introduced, followed by stepwise investigations of their physicochemical properties, functionalities, and phototoxicity.

## Materials and Methods

### Materials

Acid terminated PLGA (50:50, *M*_n_ = 7000–17000 Da), polyvinyl alcohol (PVA), dichloromethane (DCM), *N*-(3-dimethylaminopropyl)-*N*’-ethylcarbodiimide hydrochloride (EDC), sulfo-*N*-hydroxysuccinimide (Sulfo-NHS), methanol, heterobifunctional PEG (COOH-PEG-NH_2_, *M*_n_ = 3500 Da), and ICG were all purchased from Sigma-Aldrich (St. Louis, MO). Anti-HER2 monoclonal antibody (Anti-HER2-mAb) and anti-mouse immunoglobulin G (IgG) secondary antibody were purchased from Cell Signaling (Danvers, MA). All chemicals were used as received.

### Fabrication of HIPPNPs

The ICG-encapsulated PLGA nanoparticles (IPNPs) were first prepared by a modified emulsification in association with solvent evaporation method. Briefly, 1-mL methanol/DCM (v/v = 3: 7) mixture containing 1-mg ICG and 30-mg PLGA was added dropwise into 15 mL of PVA solution (0.2%, w/v) and sonicated with 20 kHz and 100 W for 90 sec in an ice bath. The emulsified solution was then stirred for another 4 h under ambient temperature to allow organic solvent evaporation. The nanoparticles (i.e., IPNPs) were collected by centrifugation at 20000 × g for 20 min, followed by wash twice with PBS to remove all the unconjugated molecules in the medium. The IPNPs suspended in 1-mL PBS were immediately subjected to surface modification using heterobifunctional PEG.

The conjugation of PEG on the surface of IPNPs was carried out by carboxyl-amine crosslinking reaction. Briefly, the IPNPs were first mixed with EDC and Sulfo-NHS (molar ratio = 2: 1) in total 2-mL PBS and incubated at room temperature in dark for 2 h. After washed twice with PBS, the collected nanoparticles were mixed with heterobifunctional PEG in total 3-mL PBS (0.2 g/mL), following incubation at room temperature in dark for 2 h. The nanoparticles were then washed twice with PBS and subjected to lyophilization afterward. The presence of PEG on the surface of IPNPs was verified by Fourier transform infrared (FTIR) spectroscopy.

**Fig 1:**
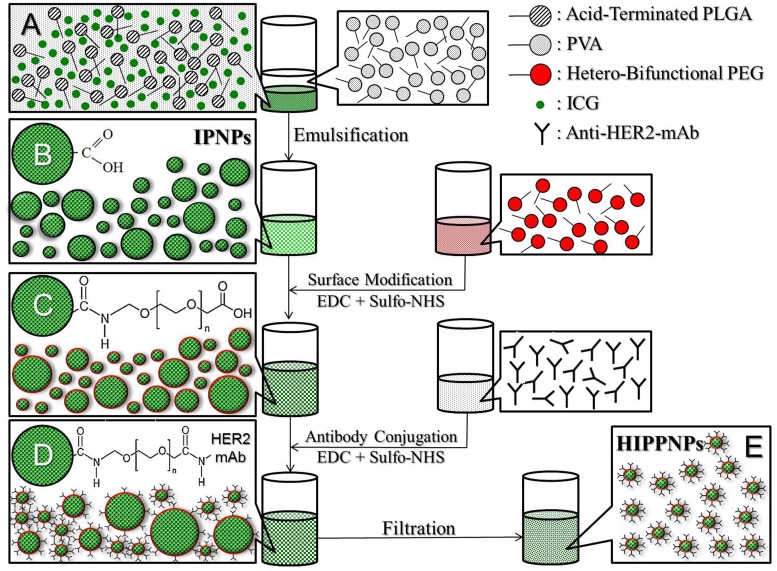
Schematic diagram of the fabrication procedures of HIPPNPs. The HIPPNPs are first formed by using a modified emulsification approach (A → B), followed by conjugation with PEG molecules (C) and then anti-HER2-mAbs (D) through EDC/Sulfo-NHS-mediated carboxyl-amine crosslinking reaction. To remove excess/unreacted chemicals and simultaneously reduce the size dispersity of the products, all the HIPPNPs with broad size distribution (D) are filtrated using a 0.45-μm filter and exhibit improved size uniformity afterward (E).

The conjugation of anti-HER2-mAbs on the surface of PEG-coated IPNPs was performed using a modified method as reported previously [28]. In brief, the lyophilized nanoparticles were first reacted with EDC and Sulfo-NHS (molar ratio = 9: 1) in PBS under ambient temperature for 2 h. After washed twice with PBS, the collected nanoparticles were then mixed with anti-HER2-mAbs in total 1-mL PBS and maintained at room temperature in dark for 1 h. To remove excess/unreacted molecules and simultaneously reduce the size dispersity of the products, the filtration method by use of a 0.45-μm filter was utilized in this study. The harvested HIPPNPs were then lyophilized for 24 h and stored at 4°C for further use. To verify the presence and activity of the anti-HER2-mAbs on the nanoparticle surface, PEG-coated IPNPs with and without anti-HER2-mAbs were separately conjugated with fluorescent anti-mouse IgG secondary antibodies and the fluorescence levels in both groups were detected by fluorescent microscopy and spectrofluorometry performed with 488 and 525 nm of excitation and emission wavelength, respectively. The overall procedures of HIPPNP fabrication is illustrated in Fig 1.

### Evaluation of physicochemical properties of HIPPNPs

The size distribution and surface charge of the HIPPNPs were measured by using dynamic light scattering (DLS) technique. The morphology of the HIPPNPs was detected by using a scanning electron microscope (S-800 SEM; HITACHI, Tokyo, Japan) with 20 kV accelerating voltage. The encapsulation efficiency of ICG (*E*_I_) was calculated by the formula:

$$E_{I}=\frac{M-M_{s}}{M}\times 100\%$$ (1)

Where *M* is total amount of ICG used for HIPPNP manufacture and *M*_*s*_ denotes the amount of unencapsulated ICG remaining in the supernatant. Both *M* and *M*_*s*_ are determined by UV-Vis spectrometry according to Beer-Lambert’s law. The percentage of ICG content in the HIPPNP (*C*_I_; wt%) was evaluated by the formula:

$$C_{I}=\frac{W_{ICG}}{W_{NP}}\times 100\%$$ (2)

Where *W*_NP_ is total weight of HIPPNPs and *W*_ICG_ denotes the mass of ICG encapsulated in the HIPPNPs tested (~ *M* × *E*_I_). The quantity of antibody on the HIPPNPs was evaluated by using the Pierce BCA Protein Assay Kit (Thermo Fisher Scientific, Waltham, MA) in association with UV-Vis spectrophotometry (λ_abs_ = 562 nm) according to the manufacturer’s instruction. The absorbance obtained from the IPPNPs was employed as the background.

$$\text{The antibody conjugation efficiency }\left(\%\right)=\frac{\text{The weight of decorated antibody }(\mu \text{g})}{\text{The weight of HIPPNPs examined }(\text{mg})}$$ (3)

#### Cell culture

The MCF7 cells (HER2-negative human breast adenocarcinoma cell line; American Type Culture Collection; ATCC, Manassas, VA) were cultured by Eagle’s minimum essential medium supplemented with 10% fetal bovine serum (FBS), 2 mM L-glutamine, 1.5 g/L sodium bicarbonate, 0.1 mM non-essential amino acids, 1.0 mM sodium pyruvate, 0.01 mg/mL bovine insulin, and 100 U/mL penicillin/streptomycin, and maintained in a 37°C incubator balanced with 5% CO_2_ and 100% humidity. The MDA-MB-453 cells (HER2-positive human breast metastatic carcinoma cell line; ATCC) were cultured by Leibovitz's L-15 medium supplemented with 10% FBS, 2 mM L-glutamine, and 100 U/mL penicillin-streptomycin, and maintained in a 37°C incubator without CO_2_.

### Analysis of degradation kinetics of ICG encapsulated in HIPPNPs

To examine the photo and thermal degradation kinetics of HIPPNP-entrapped ICG, a defined amount of HIPPNP in 9-mL PBS was aliquoted into 18 tubes and following the six were heated with 37°C in dark and the other 12 were maintained in 4°C. The six of 12 samples set in 4°C were exposed to light with intensity of 30 μmol photons/m^2^/s, while the other six were wrapped in foil to prevent light illumination. After treated for 2, 4, 6, 24, and 48 h, the HIPPNPs in all settings were washed twice with PBS and subjected to spectrophotometry (λ_abs_ = 780 nm) to analyze the amount of ICG remaining in the nanoparticles. The ICG freely dissolved in PBS with above settings were employed as the controls. The degradation rate coefficient (*k*_d_) of ICG in each group was determined based on the dynamic method [29]:

$$\frac{C_{t}}{C_{0}}=\text{exp}(-k_{d}\times t)$$ (4)

Where *C*_0_ and *C*_t_ denote the concentrations of ICG in the HIPPNPs or PBS (control) at time *t* = 0 and specific time *t* > 0, respectively.

### Examination of target specificity of HIPPNPs

Since nanoparticles with targeting ligands could be efficiently delivered into cytoplasm of targeted cells [30], the target specificity of HIPPNPs was determined by comparing the efficiencies of cellular uptake of HIPPNPs in MDA-MB-453/HER2(+) and MCF7/HER2(-) cells. For each type of cell, 3 × 10^6^ cells were aliquoted into three wells of a 24-wells culture plate and incubated at 37°C for 24 h. The HIPPNPs were added to two of three wells (1.25 μM ICG equivalent) and followed by incubation at 37°C for 2 and 4 h, respectively. The well without a HIPPNP was used as the control. At each time point, the HIPPNPs were removed by PBS wash and the fluorescent intensities of the cells were detected by spectrofluorometry performed with 750 and 838 nm of excitation and emission wavelength, respectively. In this study, the intensity of fluorescence was quantitatively represented by relative fluorescence units (RFUs) and the efficiency of cellular uptake of HIPPNPs in each setting was evaluated after normalizing the RFUs to the control.

### Analysis of cytotoxicity of HIPPNPs without light illumination

To examine the cytotoxicity of HIPPNPs in the absence of light illumination, 12-mL culture medium containing 6 × 10^6^ MDA-MB-453 cells were aliquoted into 12 wells of a 24-wells culture plate and incubated at 37°C for 24 h. Cells were then added with HIPPNPs in 0- (i.e., no HIPPNP), 1.25-, 2.5-, 5-, 10-, and 25-μM ICG equivalent and one concentration was for two wells. The viability of cells with each dose of HIPPNPs was measured after 24 and 48 h using hemocytometry with trypan blue exclusion method. In addition, the numbers of cells treated with 0, 2.5, and 25 μM ICG equivalent of HIPPNPs were further measured for seven days to establish their growth curves. The specific growth rate of the cells (*μ*) was calculated by the formula:

$$\mu \times \left(t_{2}-t_{1}\right)=\text{ln}\left(\frac{C_{t2}}{C_{t1}}\right)$$ (5)

where *C*_*t*1_ and *C*_*t*2_ denote the cell concentrations obtained at the defined time points *t*_1_ and *t*_2_, respectively.

### Measurement of singlet oxygen production of HIPPNPs

The production of singlet oxygen generated from the HIPPNPs upon light illumination was measured by using singlet oxygen sensor green (SOSG; Life Technologies, Carlsbad, CA) as the fluorescent probe. According to the manufacturer manual, 200-μL PBS containing HIPPNPs with 0 (no HIPPNP), 1.25, 2.5, 5, 10, and 25 μM ICG equivalent were separately mixed with 200-μL SOSG (10 μM), followed by incubation at room temperature in dark for 10 min. 200 μL of each sample was then transferred into one well of a 96-wells culture plate and irradiated by using a 808-nm continuous wave (CW) laser with intensity of 6 W/cm^2^. The level of SOSG-induced fluorescence in each group was detected by using a spectrofluorometer performed with 488 and 525 nm of excitation and emission wavelength, respectively, every 60 sec for 5 min. The productions of singlet oxygen generated from the freely dissolved ICG in PBS with 2.5 and 25 μM were treated as the controls.

### Measurement of HIPPNP-induced temperature increase

To evaluate the photothermal effect of HIPPNPs upon light illumination, 200-μL PBS containing HIPPNPs with 0 (no HIPPNP), 1.25, 2.5, 5, 10, and 25 μM ICG equivalent were separately irradiated by using a 808-nm CW laser with intensity of 6 W/cm^2^ for defined minutes in one well of a 96-wells culture plate. The temperature of each group was recorded every 30 sec for 5 min by using a digital thermometer. The temperature changes in 2.5- and 25-μM ICG solutions during NIR laser irradiation were employed as the controls.

### Evaluation of phototoxicity of HIPPNPs to breast cancer cells

To evaluate the phototherapeutical capacity of the HIPPNPs, 2.4-mL culture medium containing 1.2 × 10^6^ MDA-MB-453 cells were aliquoted into 12 wells of a 96-wells culture plate and incubated at 37°C for 24 h. Cells were separately added with free ICG or HIPPNPs in six wells and the ICG doses for each group were set by 0, 1.25, 2.5, 5, 10, and 25 μM. After incubation at 37°C for 4 h, all cells were washed twice with PBS and irradiated by using a 808-nm CW laser with intensity of 6 W/cm^2^ for 5 min. The cell viability in each well was assessed by using hemocytometry with trypan blue exclusion method and calcein-AM / propidium iodide (concentration ratio = 2:3) staining assay immediately after NIR irradiation.

### Statistical analysis

All data were acquired from three independent experiments and presented as mean ± standard deviation (SD). Statistical analyses were conducted by using MedCalc software in which comparisons for one condition between two groups were performed by Student’s *t*-test with a significance level of *P* < 0.05 throughout the study.

## Results and Discussion

### Verification of PEG and anti-HER2-mAbs conjugations

**Fig 2:**
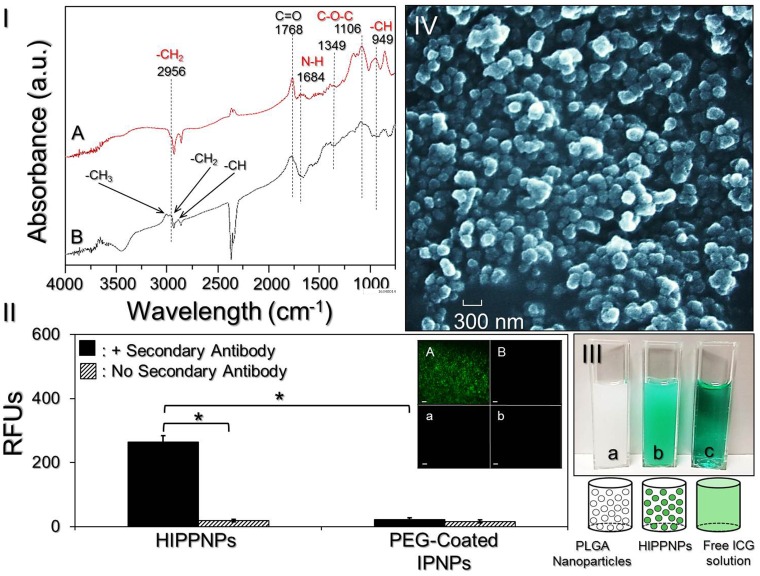
Assessment of physicochemical properties of HIPPNPs. (I) The FTIR absorption spectra of IPNPs with (A) and without (B) PEG conjugation on the nanoparticle surface. (II) Verification of presence and bioactivity of anti-HER2-mAbs on the HIPPNPs. The inset photographs represent fluoromicroscopic images of HIPPNPs (A/a) and PEG-coated IPNPs (B/b) with (A/B) and without (a/b) conjugation of fluorescent anti-mouse IgG secondary antibody at 200X magnification. Scale bar = 10 μm. The intensity of fluorescence expressed in each group was measured by spectrofluorometry performed with excitation wavelength of 488 nm and emission wavelength of 525 nm, and quantitatively represented by RFUs. Values are mean ± SD (n = 3). *P < 0.05. (III) Photographs and schematic diagrams of blank PLGA nanoparticles in PBS (a), HIPPNPs after washed with PBS (b), and freely dissolved ICG in PBS (c). (IV) SEM image of the HIPPNPs.

The presence of PEG molecules on the nanoparticle surface was verified by analyzing the characteristic peaks of the IPNPs with and without PEG conjugation through FTIR spectroscopy. As shown in Fig 2I, a strong absorbance peak at 1768 cm^−1^ corresponding to carbonyl stretching frequency of PLGA can be observed in both IPNPs with (Fig 2I; A) and without (Fig 2I; B) PEG coating. Two absorbance peaks at 1106 and 1349 cm^-1^ in the spectrum of PEG-coated IPNPs (Fig 2I; A) represent C-O-C stretching and asymmetric stretching, respectively, that indicate the presence of PEG in the sample [31]. The peak at 1684 cm^-1^ corresponding to stretching of N-H bond only in the PEG-coated IPNPs (Fig 2I; A) implicates that the carbonyl amide linkage (CONH) was successfully generated through carboxyl-amine crosslinking reaction. Furthermore, two absorbance peaks at 2956 cm^-1^ and 949 cm^-1^ corresponding to -CH_2_ stretching and -CH out-of-plane bending vibration, respectively, in the PEG-coated IPNPs (Fig 2I; A) provide another evidences for the presence of PEG in the sample [31,32]. Overall these results confirm that the PEG conjugation on the IPNP surface was successfully carried out.

The presence and activity of anti-HER2-mAb on the nanoparticle surface were examined by conjugating the particles (i.e., HIPPNPs) with the fluorescent secondary antibody and the results are shown in Fig 2II. Our data show that the HIPPNPs displayed fluorescent expression after treated with the secondary antibody (Fig 2II; inset image A) and the level of fluorescence is 14-fold (*P* < 0.05) higher than that obtained from the HIPPNPs without secondary antibody (Fig 2II, inset image a). For the PEG-coated IPNPs with secondary antibody (Fig 2II; inset image B), none of fluorescent expression is observed and the level of fluorescence is similar with that obtained from the same sample without treatment of secondary antibody (Fig 2II; inset image b, *P* = NS). These results clearly show that the HIPPNPs rather than PEG-coated IPNPs had affinity to the secondary antibody, indicating that the anti-HER2-mAbs were certainly bound on the surface of HIPPNPs and their bioconjugate activity was maintained after the carboxyl-amine crosslinking procedures.

### Characterization of HIPPNPs

Fig 2III (a)–(c) exhibits the photographs of blank PLGA nanoparticles, HIPPNPs, and freely dissolved ICG in which the green emulsified appearance of HIPPNPs (Fig 2III; b) clearly illustrates their configuration of ICG payload as compared with the other two samples. The SEM image of HIPPNPs (Fig 2IV) shows that the products maintained intact particulate morphology without collapse after the fabrication procedures including high-speed centrifugation and agitation. The mean size and surface charge of the HIPPNPs are 307 ± 4.6 nm and -17.3 ± 0.26 mV, respectively, while the polydispersity index is in the range of 0.08–0.14 after filtration. In addition, the ICG encapsulation efficiency and percentage of ICG content in the HIPPNPs are approximately 75% and 2.6 wt%, respectively, which were determined based on the Eqs (1) and (2) as described above. The conjugation efficiency of anti-HER2-mAb on the HIPPNPs is about 18 ± 0.64% according to the BCA-mediated protein analysis.

### Photo and thermal stabilities of HIPPNP-entrapped ICG

**Fig 3:**
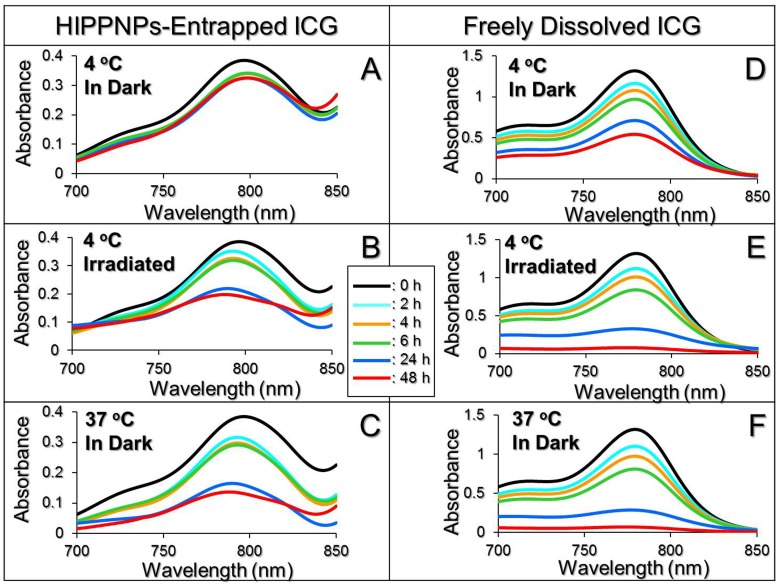
Photo and thermal degradation profiles of HIPPNP-entrapped ICG and freely dissolved ICG in PBS. A—F represent UV-Vis spectra of HIPPNPs (A—C) and freely dissolved ICG in PBS (D—F) treated with 4 or 37°C in the presence and absence of 30 μmol-photons/m^2^/s light illumination for 48 h as indicated in each figure. The peak at λ = 780 nm in each spectrum denotes the amount of ICG remaining in the HIPPNPs (A—C) or in the PBS (D—F) at 0, 2, 4, 6, 24, and 48 h during photo and/or thermal stimulation.

Fig 3 exhibits the degradation profiles of HIPPNP-entrapped ICG (Fig 3A–3C) and freely dissolved ICG in PBS (Fig 3D–3F) under maintenance at 4 (Fig 3A–3D) or 37°C (Fig 3C–3F) in dark, or under light exposure with intensity of 30 μmol-photons/m^2^/s at 4°C (Fig 3B–3E) for 48 h. As compared the degradation percentages between two settings (Table 1), our data show that the anti-degradability of ICG after entrapped into HIPPNPs significantly enhanced 3.6 folds (*P* < 0.05) with neither photo nor thermal stimulation (Fig 3A vs. 3D), while that increased 2 (*P* < 0.05) and 1.5 (*P* < 0.05) folds under lighting (Fig 3B vs. 3E) or heating (Fig 3C vs. 3F) treatment, respectively. Moreover, based on the *K*_d_ analyses (Table 1), the photostability of the HIPPNP-entrapped ICG under illumination with intensity of 30 μmol-photons/m^2^/s significantly enhanced 4 folds (*P* < 0.05) while its thermal stability under 4 and 37°C significantly enhanced 5 (*P* < 0.05) and 3 (*P* < 0.05) folds, respectively, as compared to the free ICG under equal treatment within 48 h. These outcomes demonstrate that both photo and thermal stabilities of ICG can be markedly improved after entrapped into the HIPPNPs.

**Table 1:** Degradation percentages and degradation rate coefficients of HIPPNP-entrapped ICG and freely dissolved ICG in PBS under various treatments for 48 h.

Group	% ICG degraded	*k*_d_ (h^-1^)
HIPPNP-entrapped ICG		
4°C in dark	16.3%^b^	0.0045^b^
4°C with light illumination^a^	47.1%^b^	0.0155^b^
37°C in dark	62.4%^b^	0.0233^b^
Freely dissolved ICG in PBS		
4°C in dark	58.8%	0.0206
4°C with light illumination^a^	94.3%	0.0598
37°C in dark	95.2%	0.0627

^a^Light illumination was performed by using a daylight fluorescent lamp with intensity of 30 μmol-photons/m^2^/s.

^b^*P* < 0.05 as compared to the group with freely dissolved ICG in PBS under equal illumination and/or heating treatment.

### Target specificity of HIPPNPs to HER2(+) breast cancer cells

**Fig 4:**
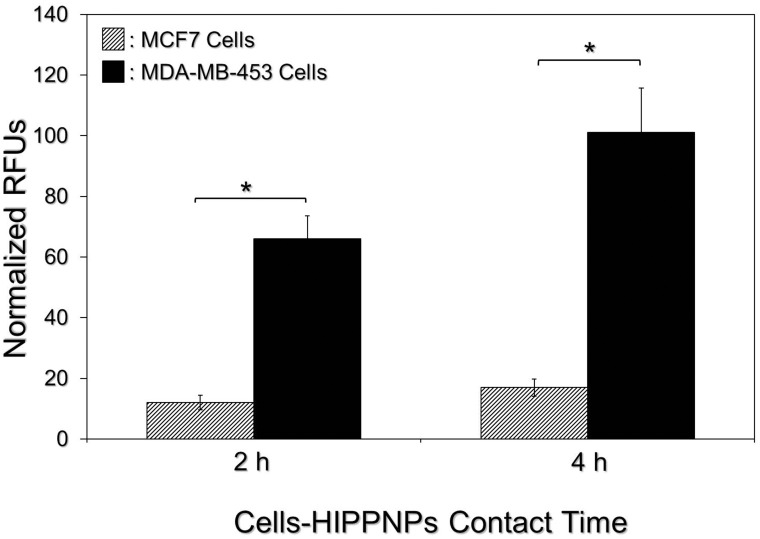
Assessment of HER2-target specificity of the HIPPNPs. After treated by HIPPNPs with 1.25 μM ICG equivalent for 2 or 4 h, the levels of fluorescence expressed from MCF7 and MDA-MB-453 cells were detected immediately after the HIPPNPs were removed. The intensity of fluorescence was detected by spectrofluorometry performed with 750 and 838 nm of excitation and emission wavelength, respectively, and quantitatively represented by RFUs after normalization to the control signal. Values are mean ± SD (n = 3). *P < 0.05.

Fig 4 exhibits the levels of ICG-induced fluorescence in MDA-MB-453 (HER2(+)) and MCF7 (HER2(-)) cells which were treated with equal amount of HIPPNPs for different time and these results represent the cellular uptake efficiencies of HIPPNPs in different types of the cells. Based on analysis of fluorescent intensity, the normalized RFUs expressed from the MDA-MB-453 cells are 5- (*P* < 0.05) and 6- (*P* < 0.05) fold higher than that obtained from the MCF7 cells after treated with HIPPNPs for 2 or 4 h, respectively. We reason that the discriminated cellular uptake efficiency of HIPPNPs is attributed to different endocytic pathways in two types of the cells. Due to expressions of anti-HER2-mAbs and HER2 receptors on the surfaces of HIPPNPs and MDA-MB-453 cells, the HIPPNPs were internalized by MDA-MB-453 cells mainly through HER2 receptor-mediated endocytosis. In terms of HER2-negative MCF7 cells, we surmise that the mechanism of HIPPNP internalization was performed by adsorptive endocytosis since it has been demonstrated as an efficient means for cancer cells to engulf negatively charged PLGA nanoparticles [33]. Receptor mediated endocytosis has been known to be more efficient and specific in comparison to adsorptive endocytosis [34], leading to a higher uptake efficiency of HIPPNPs in MDA-MB-453 cells than in MCF7 cells. These results clearly show that the HIPPNPs can be more efficiently internalized by MDA-MB-453/HER2(+) cells and whereby the target specificity of HIPPNPs to HER2-positive cells is demonstrated.

### Cytotoxicity of HIPPNPs without light illumination

**Fig 5:**
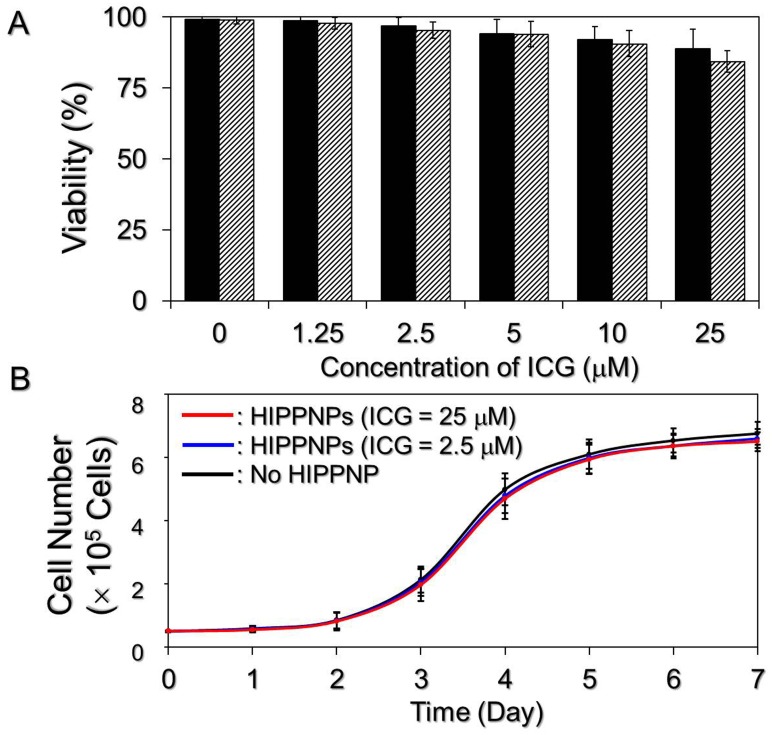
Cytotoxicity of HIPPNPs to breast cancer cells in the absence of light illumination. (A) Viabilities of MDA-MB-453 cells after treated with HIPPNPs in 0 (no HIPPNP), 1.25, 2.5, 5, 10, and 25 μM ICG equivalent without light illumination for 24 (solid) and 48 (stripe) h. The cellular viability of each group was measured by hemocytometry with trypan blue exclusion method. Values are mean ± SD (n = 3). (B) Growth kinetic curves of MDA-MB-453 cells after treated with HIPPNPs. The cells treated by HIPPNPs with 0, 2.5, and 25 μM ICG equivalent were continuously cultivated at 37°C after the viability test and the growth kinetic curve of each group was established through the measurements of cell number every 24 h for 7 days. Values are mean ± SD (n = 3).

The bioeffects of HIPPNPs on HER2-positive breast cancer cells were evaluated based on the viability and growth kinetics of MDA-MB-453 cells after treated with HIPPNPs. As shown in Fig 5A, the viabilities of cells treated by HIPPNPs with up to 25 μM ICG equivalent are all higher than 80% within 48 h. Furthermore, according to the growth kinetics study as shown in Fig 5B, the specific growth rates of the cells after treated with 2.5 and 25 μM ICG equivalent of HIPPNPs for 7 days are 0.511 and 0.509 day^-1^ respectively, and both are almost the same as the one without HIPPNP treatment (*μ* = 0.514 day^-1^). These results indicate that the HIPPNP is nontoxic to cells in the absence of light illumination.

### Photodynamic and photothermal effects of HIPPNPs

**Fig 6:**
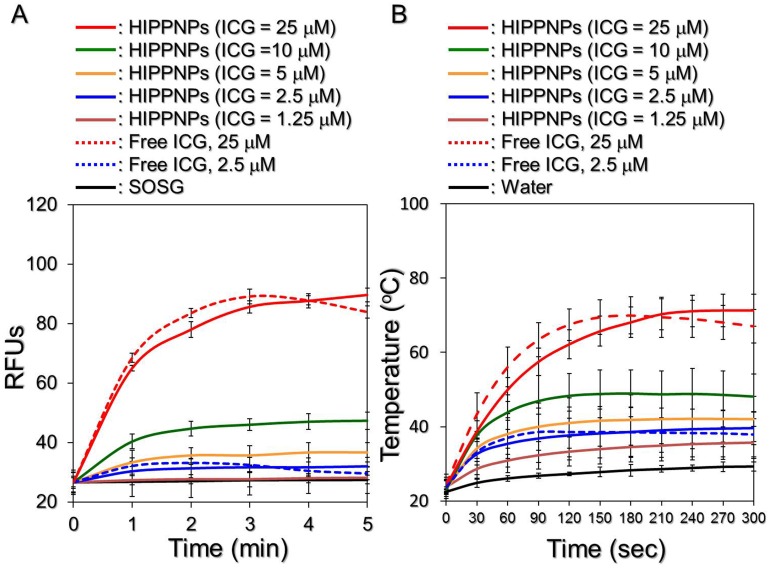
Yield of singlet oxygen and hyperthermia effect of HIPPNPs under NIR laser irradiation. Upon exposed to 808-nm CW laser with intensity of 6 W/cm^2^, both production of singlet oxygen (A) and variation of temperature (B) generated from the HIPPNPs with 0, 1.25, 2.5, 5, 10, and 25 μM ICG equivalent and freely dissolved ICG with concentrations of 2.5 and 25μM were measured every 60 (for yield of singlet oxygen) or 30 (for temperature change) sec for 5 min. The quantity of singlet oxygen was represented as the intensity of SOSG-induced fluorescence which was measured by spectrofluorometry with 488 and 525 nm of excitation and emission wavelength, respectively. The temperature at each time point was measured by using a digital thermometer. Values are mean ± SD (n = 3) in both examinations.

Fig 6A exhibits the profiles of singlet oxygen generation as presented by RFUs for the HIPPNPs and freely dissolved ICG with various concentrations upon 808-nm laser irradiation with intensity of 6 W/cm^2^. Our data show that the amount of singlet oxygen in each group increased along with NIR irradiation and a 1.1-, 1.2-, 1.4-, 1.8-, and 3.4-fold enhancement of RFU can be obtained from the HIPPNPs with 1.25, 2.5, 5, 10, and 25 μM ICG equivalent, respectively, after NIR laser treatment for 5 min. In terms of the groups using free ICG with concentrations of 2.5 and 25 μM, the fluorescence levels in both groups increased in the first 3 min of NIR laser irradiation and decreased thereafter, yielding a 1.1- and 3.2-fold enhancement of RFU, respectively, after 5-min NIR exposure. These results indicate that the HIPPNPs enable to constantly provide photodynamic effect and produce similar level of singlet oxygen within 5 min of NIR irradiation as compared to the free ICG with equal concentration.

Similar results can be found in Fig 6B; the profiles of hyperthermia effect for HIPPNPs and freely dissolved ICG with various concentrations upon NIR laser irradiation (808 nm; 6 W/cm^2^). Our data show that the temperature in each group increased along with NIR exposure and an increase of 5.1, 7.7, 13, 18.1, and 40.4°C can be obtained from the HIPPNPs with 1.25, 2.5, 5, 10, and 25 μM ICG equivalent, respectively, after 5-min NIR exposure. In terms of the groups using free ICG with concentrations of 2.5 and 25 μM, the temperatures in both settings increased in the first 90–150 sec of NIR laser irradiation and declined thereafter, yielding an increase of 7.3 and 35.6°C, respectively, after 5-min NIR laser treatment. These results indicate that the HIPPNPs enable to constantly generate hyperthermia effect within 5-min NIR laser irradiation in which the level of temperature elevation is similar with that yielded from free ICG under the same treatment.

Why the production of singlet oxygen and effect of hyperthermia in the group with free ICG reduced after 2–3 min of NIR laser irradiation? We surmise that it was because ICG degradation in aqueous medium can be accelerated by light and heat [20], the ICG molecules freely dissolved in PBS continuously lost their ability to generate singlet oxygen and heat during NIR irradiation and resulted in declines of RFU and temperature (Fig 6) when the generation efficiency of singlet oxygen and/or heat was lower than their depletion rate in the medium. On the contrary, the photodynamic and photothermal effects of HIPPNPs were lasted since the photo and thermal stabilities of entrapped ICG remarkably enhanced due to protection of polymeric matrix as analyzed in Table 1.

The temperature level has been known to play a key role in the efficacy of photothermal therapy. According to the cellular assays as reported previously, irreversible cell damage can be obtained after heated at 40–45°C for 30–60 min [35], while only 4–6 min is sufficient at 50–52°C [36,37]. At > 60°C, the time that is required to cause irreversible cell damage decreases exponentially because denaturation of cytoplasmic proteins and/or enzymes rapidly occurs and leads to coagulated necrosis immediately [38]. Although higher temperature may provide more opportunities to eradicate tumor cells, operating temperature in the range of 41–43°C is frequently utilized in the clinic to minimize the potential deleterious influence on the surrounding normal cells [39,40]. Based on the results shown in Fig 6, we hypothesize that HIPPNPs with ≥ 5 μM ICG equivalent are able to provide both photodynamic and hyperthermia (T > 40°C) effects for tumor destruction, whereas the effect of phototherapy of HIPPNPs with ≤ 2.5 μM ICG equivalent will be majorly dependent on the photodynamic efficacy under 808-nm CW laser irradiation with intensity of 6 W/cm^2^ for 5 min.

### Phototoxic effect of HIPPNPs on HER2(+) and HER2(-) breast cancer cells

**Fig 7:**
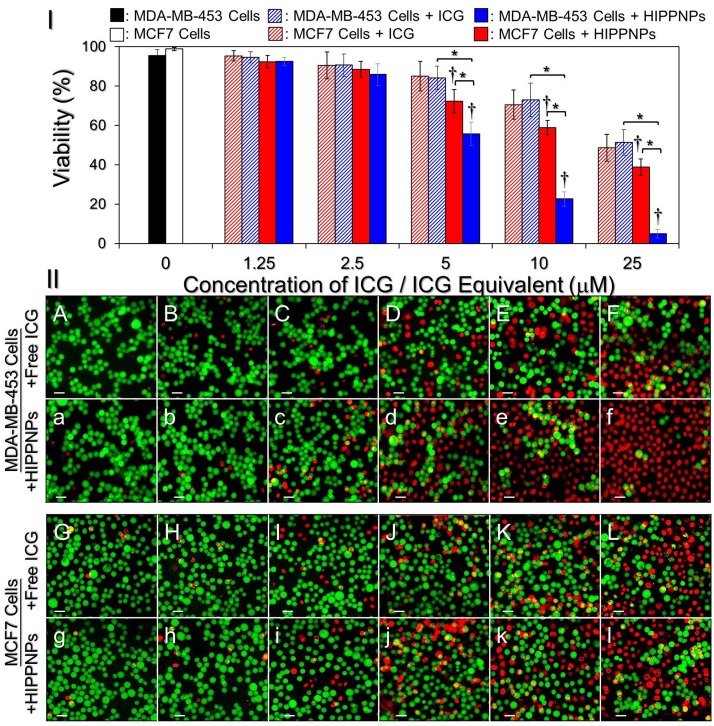
Phototoxicity of free ICG and HIPPNPs to HER2(+) and HER(-) breast cancer cells *in vitro*. (I) Viabilities of MDA-MB-453 and MCF7 cells pre-treated with freely dissolved ICG or HIPPNPs in different doses and subjected to light illumination afterward. Both free ICG and HIPPNPs were exploited in concentrations of 0 (without agents; blank control), 1.25, 2.5, 5, 10, and 25 μM ICG equivalent for each type of cells and were co-cultured with the cells for only 4 h. The light illumination was performed by using a 808-nm CW laser with intensity of 6 W/cm^2^ for 5 min after the free ICG or HIPPNPs were removed. The cellular viability was determined by hemocytometry with trypan blue exclusion method immediately after the laser treatment. Values are mean ± SD (n = 3). *P < 0.05. ^†^*P* < 0.05 as compared to the blank control. (II) Representative photomicrographic images of calcein-AM/PI-stained MDA-MB-453 (A/a–F/f) or MCF7 (G/g–L/l) cells after NIR laser irradiation. Before NIR illumination, cells were treated with free ICG (A—L) or HIPPNPs (a—l) in 0 (A/a and G/g), 1.25 (B/b and H/h), 2.5 (C/c and I/i), 5 (D/d and J/j), 10 (E/e and K/k), and 25 (F/f and L/l) μM ICG equivalent for 4 h and followed by PBS wash. The green cells stained by calcein-AM and the red cells stained by PI represent live and dead cells, respectively. All stained cells were suspended in PBS and the images were photographed by using a fluorescent microscope at 200X magnification. Scale bar = 50 μm.

We subsequently examined the phototoxicity of HIPPNPs in different doses to MDA-MB-453 and MCF7 cells under NIR laser irradiation and the results of cellular viabilities determined by using hemocytometry and calcein-AM/PI staining assay are shown in Fig 7I and 7II, respectively. Our data show that the viabilities of NIR laser-treated MDA-MB-453 and MCF7 cells without ICG are 96% (Fig 7II; A/a) and 99% (Fig 7II; G/g), respectively, indicating that the slight temperature increase due to NIR laser irradiation (Fig 6B) is nontoxic to cells. Both free ICG and HIPPNPs provided dose-dependent toxicity to the cells, and the HIPPNP-induced cytotoxicity was higher than that caused by free ICG throughout the does range in both types of cells (Fig 7II; B–F vs. b–f, G–L vs. g–l). In terms of HIPPNP-treated groups, our data showed that the HER2-positive MDA-MB-453 cells underwent severer cell death upon NIR irradiation (808 nm, 6 W/cm^2^) as compared with HER2-negative MCF7 cells that the viabilities in the former cells were 1.03-, 1.12-, 1.3- (*P* < 0.05), 2.6- (*P* < 0.05), and 8-fold (*P* < 0.05) lower the latter ones while the concentrations of HIPPNP-entrapped ICG utilized for the phototherapy were 1.25, 2.5, 5, 10, and 25 μM, respectively. Moreover, we found that the phototoxicity of the HIPPNPs, rather than free ICG, to the MDA-MB-453 cells substantially enhanced since the dose of HIPPNPs was ≥ 5 μM ICG equivalent that the viability was significantly reduced 11-fold (*P* < 0.05) while the concentration of entrapped ICG was increased from 5 to 25 μM.

According to the results shown in Fig 4, we speculate that the reason why the HIPPNPs were more toxic to MDA-MB-453 cells than MCF7 cells is because HIPPNPs can be more efficiently internalized/absorbed by MDA-MB-453/HER2(+) cells due to HER2-target specificity of the HIPPNPs and thereby led to a higher efficacy of cell eradication for the groups with MDA-MB-453 cells. In addition, based on the results shown in Fig 6, we reason that the significant phototoxicity of HIPPNPs with ≥ 5 μM ICG equivalent was achieved due to combination of photodynamic and photothermal effects, while the HIPPNPs with ≤ 2.5 μM ICG equivalent can only provide mild photodynamic and hyperthermia effects and thus resulted in a moderate effect of cancer cell killing.

One may question that why the phototoxicity of HIPPNPs with 25 μM ICG equivalent is significantly higher than that obtained by using free ICG (Fig 7) since they can provide similar photodynamic and hyperthermia effects within 5 min of NIR laser irradiation (Fig 6)? We speculate that it was because in this study, the free ICG and HIPPNPs were only co-cultured with the cells for 4 h and the degradation efficiency of free ICG within 4 h at 37°C was about two folds higher than HIPPNP-entrapped ICG (Fig 3C vs. 3F), implicating that the amount of intact ICG transferred by free molecules was lower than that delivered by HIPPNPs. In addition, the HIPPNPs can be efficiently internalized by MDA-MB-453 cells due to their HER2-target specificity (Fig 4) and enabled to protect the entrapped ICG from immediate degradation caused by low pH in the late endosome [27,41]. These properties significantly improved the availability of HIPPNPs for use in phototherapy and thus resulted in a significantly enhanced efficacy of cancer cell killing in comparison to free ICG.

## Conclusions

In this study, we have successfully manufactured HIPPNPs, a type of HER2-specific biodegradable NIR photosensitive agents, for targeted phototherapy of HER2-positive breast cancer cells. We not only investigated their physicochemical properties and functionalities in respect of generation of singlet oxygen and hyperthermia efficacy upon NIR laser irradiation, but also demonstrated the availability of HIPPNPs in different doses for eradication of tumor cells *in vitro* through use of HER2-positive MDA-MB-453 breast cancer cells. In addition to the merits of HIPPNPs as described above, the PEG molecules conjugated on the outer layer of HIPPNPs may render the nanoparticles a prolonged circulation time in the bloodstream due to less immunogenicity of PEG [26], and such characteristics is particularly favorable for passive targeting of cancer cells through enhanced permeability and retention (EPR) effect [42,43]. Therefore, we anticipate that the HIPPNPs may provide an improved therapeutic effectiveness for *in vivo* applications as compared with free ICG. To fully address the efficacy of HIPPNPs on phototherapy of breast cancer cells identified in this study, further *in vivo* study is certainly needed and efforts are currently in progress.

## References

[1] Jemal A, Bray F, Center MM, Ferlay J, Ward E, Forman D. Global cancer statistics. CA Cancer J Clin. 2011;61:69–90. 10.3322/caac.20107 21296855

[2] Tinoco G, Warsch S, Glück S, Avancha K, Montero AJ. Treating breast cancer in the 21st century: emerging biological therapies. J Cancer. 2013;4:117–132. 10.7150/jca.4925 23386910 PMC3563073

[3] Press MF, Slamon DJ, Flom KJ, Park J, Zhou JY, Bernstein L. Evaluation of HER-2/neu gene amplification and overexpression: comparison of frequently used Assay methods in a molecularly characterized cohort of breast cancer. J Clin Oncol. 2002;20:3095–3105. 12118023 10.1200/JCO.2002.09.094

[4] Hudziak RM, Schlessinger J, Ullrich A. Increased expression of the putative growth factor receptor p185HER2 causes transformation and tumorigenesis of NIH 3T3 cells. Proc Natl Acad Sci USA. 1987;84:7159–7163. 2890160 10.1073/pnas.84.20.7159 PMC299249

[5] Choudhury A, Kiessling R. Her-2/neu as a paradigm of a tumor-specific target for therapy. Breast Dis. 2004;20:25–31. 15687704 10.3233/bd-2004-20104

[6] Slamon DJ, Clark GM, Wong SG, Levin WJ, Ullrich A, McGuire WL. Human breast cancer: correlation of relapse and survival with amplification of the HER-2/neu oncogene. Science. 1987;235:177–182. 3798106 10.1126/science.3798106

[7] Leonard DS, Hill AD, Kelly L, Dijkstra B, McDermott E, O'Higgins NJ. Anti-human epidermal growth factor receptor 2 monoclonal antibody therapy for breast cancer. Br J Surg. 2002;89:262–271. 10.1046/j.0007-1323.2001.02022.x 11872048

[8] Ross JS, Slodkowska EA, Symmans WF, Pusztai L, Ravdin PM, Hortobagyi GN. The HER-2 receptor and breast cancer: ten years of targeted anti-HER-2 therapy and personalized medicine. Oncologist. 2009;14:320–368. 10.1634/theoncologist.2008-0230 19346299

[9] Fiszman GL, Jasnis MA. Molecular mechanisms of trastuzumab resistance in HER2 overexpressing breast cancer. Int J Breast Cancer. 2011;2011:352182 10.4061/2011/352182 22295219 PMC3262573

[10] Ntziachristos V, Bremer C, Weissleder R. Fluorescence imaging with near-infrared light: new technological advances that enable in vivo molecular imaging. Eur Radiol. 2003;13:195–208. 10.1007/s00330-002-1524-x 12541130

[11] Cheng L, Wang C, Feng LZ, Yang K, Liu Z. Functional nanomaterials for phototherapies of cancer. Chem Rev. 2014;114:10869–10939. 10.1021/cr400532z 25260098

[12] Dolmans DE, Fukumura D, Jain RK. Photodynamic therapy for cancer. Nat Rev Cancer. 2003;3:380–387. 10.1038/nrc1071 12724736

[13] Circu ML, AW TY. Reactive oxygen species, cellular redox systems, and apoptosis. Free Radic Biol Med. 2010;48:749–762. 10.1016/j.freeradbiomed.2009.12.022 20045723 PMC2823977

[14] Schaafsma BE, Mieog JS, Hutteman M, van der Vorst JR, Kuppen PJ, Löwik CW et al The clinical use of indocyanine green as a near-infrared fluorescent contrast agent for image-guided oncologic surgery. J Surg Oncol. 2011;104:323–332. 10.1002/jso.21943 21495033 PMC3144993

[15] Mastropasqua R, Di Antonio L, Di Staso S, Agnifili L, Di Gregorio A, Ciancaglini M, et al Optical Coherence Tomography Angiography in Retinal Vascular Diseases and Choroidal Neovascularization. J Ophthalmol. 2015;2015:343515 10.1155/2015/343515 26491548 PMC4600507

[16] Zelken JA, Tufaro AP. Current Trends and Emerging Future of Indocyanine Green Usage in Surgery and Oncology: An Update. Ann Surg Oncol. 2015;Suppl 3:1271–1283. 26193966 10.1245/s10434-015-4743-5

[17] Shemesh CS, Moshkelani D, Zhang H. Thermosensitive liposome formulated indocyanine green for near-infrared triggered photodynamic therapy: in vivo evaluation for triple-negative breast cancer. Pharm Res. 2015;32:1604–1614. 10.1007/s11095-014-1560-7 25407543

[18] Bernardi RJ, Lowery AR, Thompson PA, Blaney SM, West JL. Immunonanoshells for targeted photothermal ablation in medulloblastoma and glioma: an in vitro evaluation using human cell lines. J Neurooncol. 2008;86:165–172. 10.1007/s11060-007-9467-3 17805488

[19] Mundra V, Peng Y, Rana S, Natarajan A, Mahato RI. Micellar formulation of indocyanine green for phototherapy of melanoma. J Control Release. 2015;220:130–140. 10.1016/j.jconrel.2015.10.029 26482083

[20] Saxena V, Sadoqi M, Shao J. Degradation kinetics of indocyanine green in aqueous solution. J Pharm Sci. 2003;92:2090–2097. 10.1002/jps.10470 14502548

[21] Mordon S, Devoisselle JM, Soulie-Begu S, Desmettre T. Indocyanine green: physicochemical factors affecting its fluorescence in vivo. Microvasc Res. 1998;55:146–152. 10.1006/mvre.1998.2068 9521889

[22] Desmettre T, Devoisselle JM, Mordon S. Fluorescence properties and metabolic features of indocyanine green (ICG) as related to angiography. Surv Ophthalmol. 2000;45:15–27. 10946079 10.1016/s0039-6257(00)00123-5

[23] Jain KK. Nanomedicine: application of nanobiotechnology in medical practice. Med Princ Pract. 2008;17:89–101. 10.1159/000112961 18287791

[24] Nair LS, Laurencin CT. Biodegradable polymers as biomaterials. Prog Polym Sci. 2007;32:762–798.

[25] Makadia HK, Siegel SJ. Poly lactic-co-glycolic acid (PLGA) as biodegradable controlled drug delivery carrier. Polymers. 2011;3:1377–1397. 10.3390/polym3031377 22577513 PMC3347861

[26] Fishburn CS. The pharmacology of PEGylation: Balancing PD with PK to generate novel therapeutics. J Pharm Sci. 2008;97:4167–4183. 10.1002/jps.21278 18200508

[27] Björnsson OG, Murphy R, Chadwick VS, Björnsson S. Physiochemical studies on indocyanine green: molar lineic absorbance, pH tolerance, activation energy and rate of decay in various solvents. J Clin Chem Clin Biochem. 1983;21:453–458. 6619743 10.1515/cclm.1983.21.7.453

[28] Yang J, Lee C, Park J, Seo S, Lim E, Song YJ, et al Antibody conjugated magnetic PLGA nanoparticles for diagnosis and treatment of breast cancer. J Mater Chem. 2007;17:2695–2699.

[29] Saxena V, Sadoqi M, Shao J. Enhanced photo-stability, thermal-stability and aqueous-stability of indocyanine green in polymeric nanoparticulate systems. J Photochem Photobiol B. 2004;74:29–38. 10.1016/j.jphotobiol.2004.01.002 15043844

[30] Hu CM, Kaushal S, Tran Cao HS, Aryal S, Sartor M, Esener S, et al Half-antibody functionalized lipid-polymer hybrid nanoparticles for targeted drug delivery to carcinoembryonic antigen presenting pancreatic cancer cells. Mol Pharm. 2010;7:914–920. 10.1021/mp900316a 20394436 PMC2884057

[31] Sun C, Sze R, Zhang M. Folic acid-PEG conjugated superparamagnetic nanoparticles for targeted cellular uptake and detection by MRI. J Biomed Mater Res A. 2006;78:550–557. 10.1002/jbm.a.30781 16736484

[32] Gajendiran M, Yousuf SMJ, Elangovan V, Balasubramanian S. Gold nanoparticle conjugated PLGA–PEG–SA–PEG–PLGA multiblock copolymer nanoparticles: synthesis, characterization, in vivo release of rifampicin. J Mater Chem B. 2014;2:418–427. 10.1039/c3tb21113d 32261386

[33] Harush-Frenkel O, Debotton N, Benita S, Altschuler Y. Targeting of nanoparticles to the clathrin-mediated endocytic pathway. Biochem Biophys Res Commun. 2007;353:26–32. 10.1016/j.bbrc.2006.11.135 17184736

[34] Ogris M, Steinlein P, Carotta S, Brunner S, Wagner E. DNA/polyethylenimine transfection particles: influence of ligands, polymer size, and PEGylation on internalization and gene expression. AAPS PharmSci. 2001;3:E21 10.1208/ps030321 11741272 PMC2751016

[35] Chu KF, Dupuy DE. Thermal ablation of tumours: biological mechanisms and advances in therapy. Nat Rev Cancer. 2014;14:199–208. 10.1038/nrc3672 24561446

[36] Thomsen S. Pathologic analysis of photothermal and photomechanical effects of laser-tissue interactions. Photochem Photobiol. 1991;53:825–835. 1886941 10.1111/j.1751-1097.1991.tb09897.x

[37] Goldberg SN, Gazelle GS, Halpern EF, Rittman WJ, Mueller PP, Rosenthal DI. Radiofrequency tissue ablation: importance of local temperature along the electrode tip exposure in determining lesion shape and size. Acad Radiol. 1996;3:212–218. 8796667 10.1016/s1076-6332(96)80443-0

[38] Haen SP, Pereira PL, Salih HR, Rammensee HG, Gouttefangeas C. More than just tumor destruction: immunomodulation by thermal ablation of cancer. Clin Dev Immunol. 2011;2011:160250 10.1155/2011/160250 22242035 PMC3254009

[39] Hauck TS, Jennings TL, Yatsenko T, Kumaradas JC, Chan WCW. Enhancing the Toxicity of Cancer Chemotherapeutics with Gold Nanorod Hyperthermia. Advanced Materials. 2008;20:3832–3838.

[40] Coffey D, Getzenberg R, DeWeese T. Hyperthermic biology and cancer therapies: a hypothesis for the "Lance Armstrong effect". J Am Med Assoc. 2006;296:445–448. 10.1001/jama.296.4.445 16868303

[41] Sorkin A, Von Zastrow M. Signal transduction and endocytosis: close encounters of many kinds. Nat Rev Mol Cell Biol. 2002;3:600–614. 10.1038/nrm883 12154371

[42] Lammers T, Kiessling F, Hennink WE, Storm G. Drug targeting to tumors: principles, pitfalls and (pre-) clinical progress. J Control Release. 2012;161:175–187. 10.1016/j.jconrel.2011.09.063 21945285

[43] Jain RK, Stylianopoulos T. Delivering nanomedicine to solid tumors. Nat Rev Clin Oncol. 2010;7:653–664. 10.1038/nrclinonc.2010.139 20838415 PMC3065247

